# Low-frequency ultrasonic pulse-echo datasets for geometry determination of a reinforced concrete step specimen with embedded polystyrene foam cuboids in non-destructive testing

**DOI:** 10.1016/j.dib.2025.111956

**Published:** 2025-08-05

**Authors:** Maria Grohmann, Stefan Maack, Matthias Behrens, Ernst Niederleithinger

**Affiliations:** Federal Institute for Materials Research and Testing (BAM), Unter den Eichen 87, Berlin 12205, Germany

**Keywords:** Civil engineering, Concrete structures, Ultrasonic echo testing, Reference data

## Abstract

The dataset comprises raw data collected using the ultrasonic pulse-echo method on a concrete specimen. The specimen addresses two fundamental non-destructive testing (NDT) challenges in civil engineering: locating built-in elements and determining dimensions to analyse both the internal and external geometry of concrete structures.

For data acquisition, the surface of the concrete specimen was automatically scanned point by point, with pulse-echo measurements performed at each measuring point. The automation of the measurement process ensured high repeatability and precision as well as a high measurement point density. Two low-frequency probes were used, each capable of both transmitting and receiving ultrasonic waves: one for longitudinal waves and one for shear waves**.** The longitudinal wave probe operates at frequencies of up to approximately 160 kHz, whereby a frequency of 80 kHz was used for the ultrasonic measurements here. The shear wave probe operates at up to 100 kHz, with a frequency of 50 kHz used for the measurements in this study. This article includes additional technical specifications of the two ultrasonic probes used, as well as information on their geometric specifications and directivity patterns. It also provides a detailed description of the further measurement equipment used. The ultrasonic raw data are stored in a universally readable format, with each time signal (A-scan) spanning two milliseconds and a sampling rate of two mega-samples per second. The provided data support comparative studies in signal analysis, imaging as well as interpretation and can be used for evaluation purposes in various practical NDT scenarios.

Specifications Table

**Table:** 

Subject	Engineering & Materials science
Specific subject area	Automated ultrasonic echo measurements on a concrete specimen for diverse non-destructive testing (NDT) scenarios in civil engineering.
Type of data	Time series Raw data
Data collection	The ultrasonic data were collected on a concrete specimen using an automated scanner system. The specimen was produced at the Materials Testing Institute MPA in Stuttgart and is now owned by the Federal Institute for Materials Research and Testing BAM in Berlin. Automated scanning system: Development by BAM Probes: M2502 – Dual Aperture Shear Wave DPC Transducer Array and M2503 – Dual Aperture Longitudinal Wave DPC Transducer Array (Acoustic Control Systems - ACS Group) Driving signal generator: Development by BAM Data Acquisition: USB-6361 (National Instruments Corp.) Software: LabVIEW© (National Instruments Corp.) based-scanner control and data acquisition software developed by BAM
Data source location	Institution: Bundesanstalt für Materialforschung und -prüfung (Federal Institute for Materials Research and Testing), BAM City/Town/Region: Berlin Country: Germany
Data accessibility	Repository name: HARVARD Dataverse Data identification number: 10.7910/DVN/0HHJFF Direct URL to data: https://doi.org/10.7910/DVN/0HHJFF
Related research article	none

## 1 Value of the Data

• Testing concrete structures using the ultrasonic echo method poses challenges due to varying boundary conditions encountered in real-world NDT applications, such as on construction sites, where different components require examination. To address these challenges, it is essential to analyse and enhance the performance of evaluation algorithms for processing and interpreting ultrasonic data. The provided data address a significant range of common NDT scenarios.
• This data will be particularly beneficial for users of ultrasonic techniques aiming to assess and improve their evaluation methods, as well as for hardware and software developers and researchers focusing on data processing and imaging methods for pulse-echo techniques.
• Comparing data processing and imaging algorithms proposed by different authors or institutions is often difficult because they are typically demonstrated using measurements obtained from varying devices, setups, and test objects. To facilitate such comparisons, we provide well-documented data that include diverse testing scenarios and are based on different wave types, enabling robust comparison of the performance of evaluation algorithms.
• The dataset is also valuable for developing and evaluating new experimental designs based on the presented concrete test specimen.
• The dataset is well-suited for training and qualifying personnel involved in NDT methods.

## 2 Background

This paper builds upon the work of Maack et al. [2], in which ultrasonic reference data from two similar concrete specimens were published. The datasets published in this article [1] were recorded using the same ultrasonic measurement technique but on a concrete specimen with a different internal geometry. As a result, it offers researchers and practitioners working with ultrasonic echo techniques additional NDT testing scenarios to assess and improve their data processing and evaluation methods.

## 3 Data Description

**Fig. 1:**
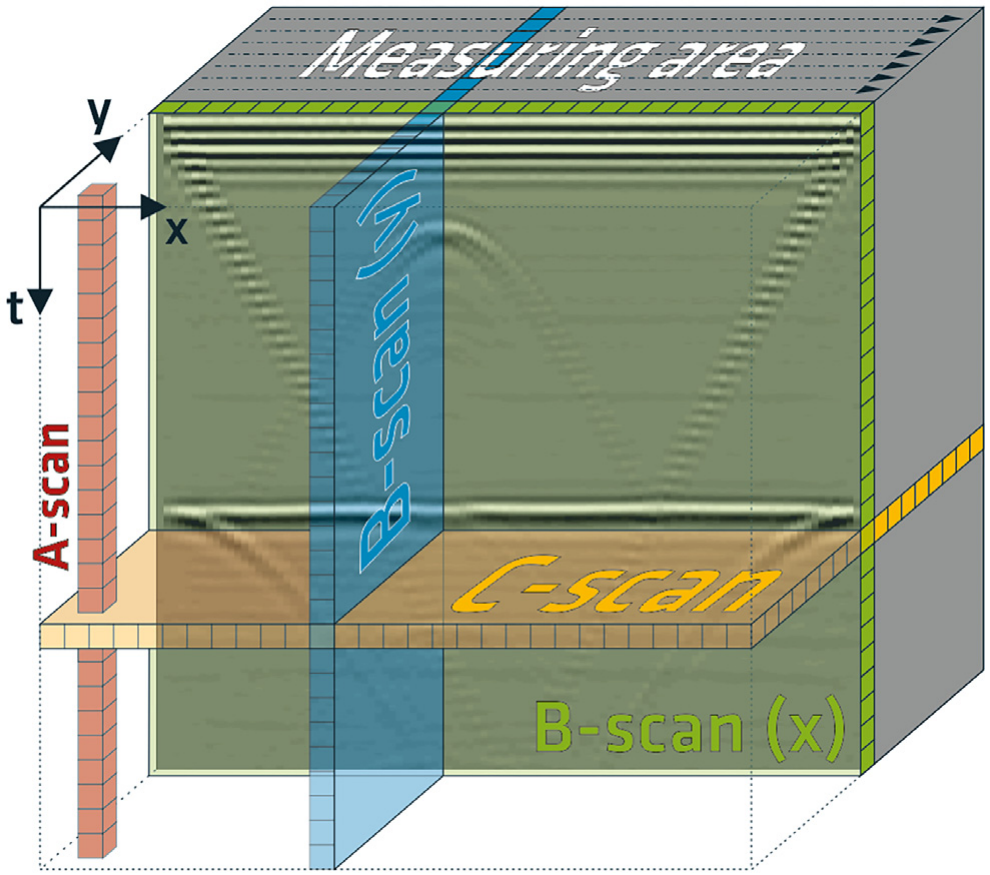
Overview of the designations of the different data that can be derived from an area measurement: Time-signal at a single measurement point (A-scan), time-signals (A-scans) sampled along a measurement line (B-scan), and depth slice through time (C-scan); extracted from [2].

The ultrasonic-echo data is raw data, which was collected point by point on a defined measurement grid on the surface of a concrete specimen. The BAM internal designation for this specimen is Pk401 (‘Pk’ is a German abbreviation for ‘Prüfkörper’, English: ‘test specimen’). At each measurement point, a pulse-echo measurement was performed, where both the transmitting and receiving probes were positioned on the same side of the specimen. The signal obtained from a single pulse-echo measurement is a time-domain signal, commonly referred to as an A-scan in ultrasonic testing [3]. An A-scan displays the sound pressure of the received signal as amplitude over the pulse's travel time. By recording A-scans at multiple points within a defined measurement area, a three-dimensional (3D) dataset containing the volume information of the investigated specimen can be generated (Fig. 1).

The used sampling rate for the ultrasonic-echo measurements was f = 2 MHz, which corresponds to a time sampling interval of Δt = 0.5 µs. Each A-scan in the dataset has a time length of t = 2000 µs, corresponding to 4000 samples. The recorded data were stored as 16-bit signed integers within the amplitude ranges A = ±1000 mV or A = ±2000 mV, depending on the type of probe used (for longitudinal or shear waves) (see section MEASUREMENT SETUP AND PROCESS, Table 3).

**Fig. 2:**
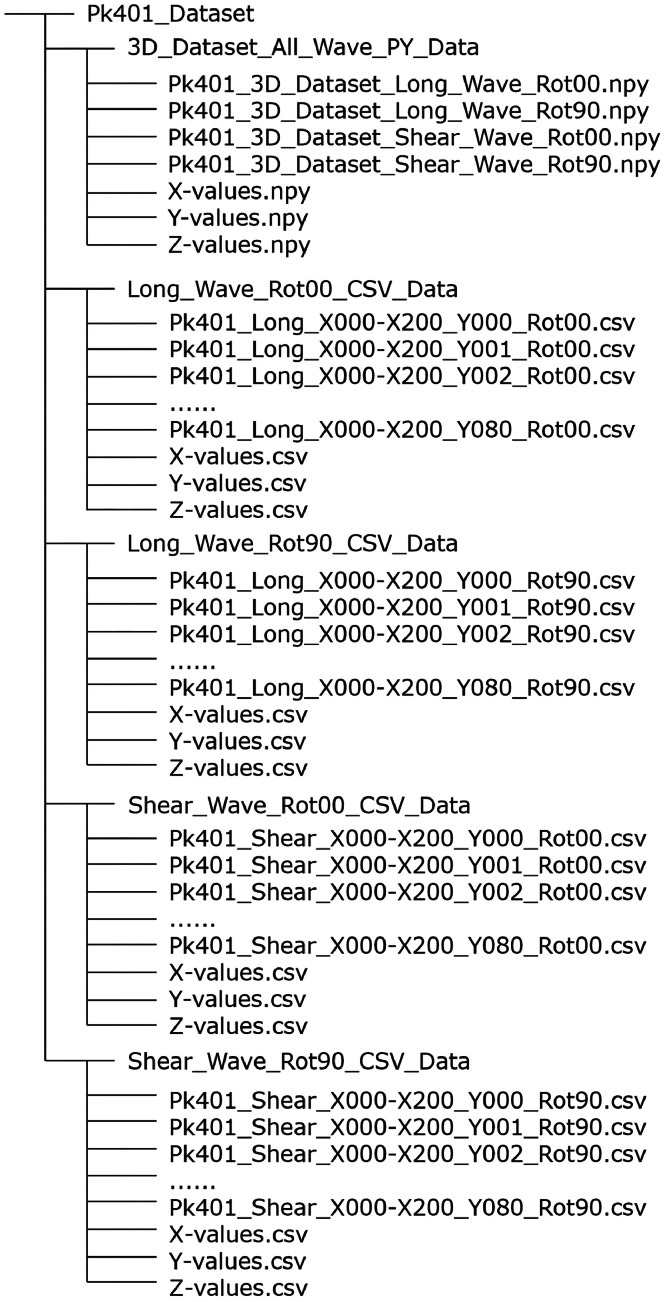
Data structure of the different file formats and data storage types.

The ultrasonic-echo data contain the raw data as well as geometrical information and are available in different formats and types. First, individual B-scans (which consist of a series of A-scans sampled consecutively along a measurement line, as shown in Fig. 1) are provided in the commonly accessible CSV format (∗.csv files) [4]. Second, the measurement data are made available in NumPy format (∗.npy files, Python version 3.11.7) in a 3D array. Fig. 2 demonstrates the data structure of the different file formats and data storage types.

**Fig. 3:**
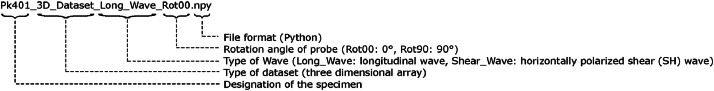
Exemplary description of the file names for the files stored in NumPy-format (∗.npy).

The subfolder *3D_Dataset_All_Wave_PY_Data* contains the 3D-datasets in NumPy format (∗.npy-files), which can be accessed in Python via the np.load("path/to/file.npy") function. The four data sets differ based on the wave type (longitudinal or shear) and the probe orientation (Rot00 or Rot90) on the concrete surface, which defines the direction of polarization. In section MEASUREMENT SETUP AND PROCESS the two different probe orientations are explained in more detail (Fig. 14). Furthermore, three additional vectors are included, comprising the geometrical orientation of the measurement points in X- and Y-directions (X-values and Y-values, in mm) and the time values for the samples (Z-values in µs). The X- und Y- axes of the measurement plane are visualized in Appendix A. The designation for the 3D datasets is explained in Fig. 3 exemplarily.

**Fig. 4:**
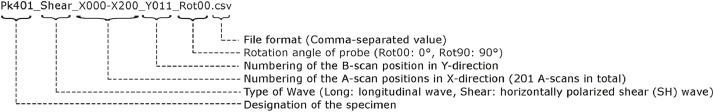
Exemplary description of the file names for the files stored in CSV-format (∗.csv).

**Fig. 5:**
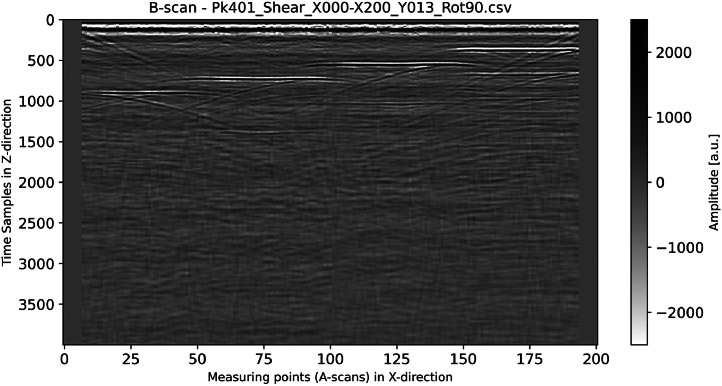
B-scan (color-coded in grayscale) generated from the dataset “Pk401_Shear_X000-X200_Y013_Rot90.csv”.

The data tree (Fig. 2) contains four additional subfolders, each storing two-dimensional (2D) B-scan datasets in CSV format (∗.csv-files). The folder names indicate the wave type (longitudinal or shear) and the probe orientation. Unlike the Python datasets, the CSV datasets are stored in individual B-scans along the X-axis of the measurement area. In addition, each folder contains three vectors: two that describe the geometrical information of the measurement points in X- and Y-directions (X-values and Y-values, in mm), and one that contains the time values in Z-direction (Z-values in µs). The designation for the 2D datasets is demonstrated in Fig. 4 exemplarily. The position of the A-scan numbered X000 is located at the origin of the coordinate system (X/Y = 0 mm), which is marked in the technical drawings in Appendix A with a circle containing a cross. The B-scan numbered Y000, which contains the A-scans X000-X200, is located at Y = 0 mm. Additionally, it should be noted that the first (Y000-Y004) and last (Y076-Y080) B-scans as well as the first (X000-X006) and last (X194-X200) A-scans of each B-scan do not contain measurement data (see zero A-scans in Fig. 5). In these areas, the probes would not have been fully positioned on the surface of the concrete specimen, as individual transducers of the probes would have been outside the measurement area. Therefore, to accurately represent the geometric dimensions of the specimen, corresponding zero vectors and matrices are provided for the A-Scans and B-Scans mentioned above. The geometric dimensions of the concrete specimen are outlined in detail in Appendix A and Appendix B, Appendix A and Appendix B.

## 4 Experimental Design, Materials and Methods

### 4.1 Concrete specimen Pk401

**Fig. 6:**
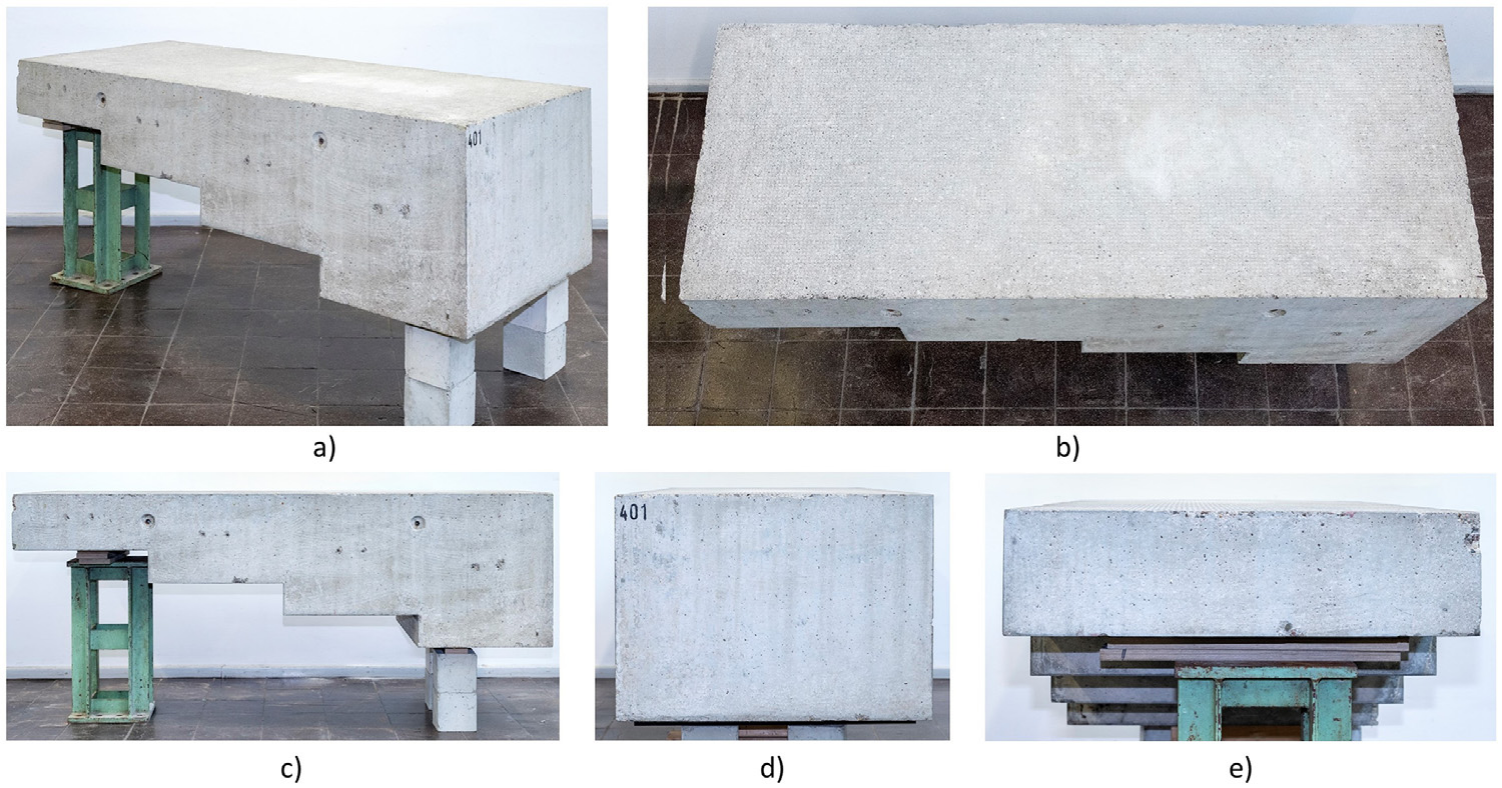
Concrete specimen Pk401: a) 3D perspective, b) top view, and c), d) lateral views.

The concrete specimen Pk401 (Fig. 6) was produced at the Materials Testing Institute MPA in Stuttgart as part of the research project „FOR 384: Zerstörungsfreie Strukturbestimmung von Betonbauteilen mit akustischen und elektromagnetischen Echo-Verfahren“ [5]. This project was funded by the German Research Foundation (Deutsche Forschungsgemeinschaft DFG) in the years 2000 to 2007 (grant number: 5466784).

**Fig. 7:**
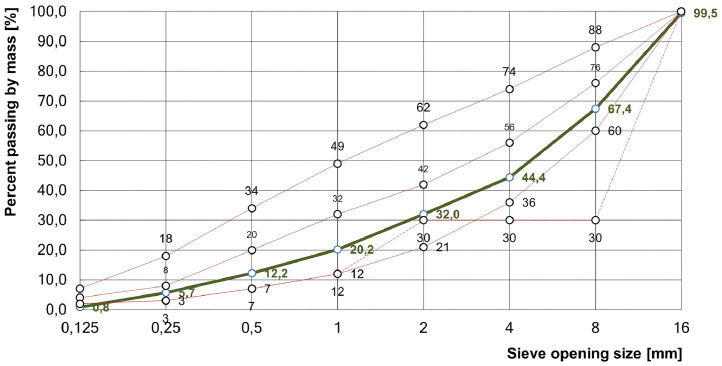
Recalculation of the grading curve A/B 16 (green solid line) (taken from [2]). The red lines show the limiting grading envelopes for a maximum aggregate size of 16 mm (GK 16) according to DIN 1045-2 [6].

The concrete step specimen was planned according to the same conceptual design as the two other concrete specimens constructed within the FOR 384 research project, which are described in detail in Maack et al. [2], and therefore features a comparable concrete mixture. The precise mixture properties, such as cement type and water-to-cement (w/c) ratio, are not known. Confirmed parameters are a maximum aggregate size of 16 mm (GK 16) and a compressive strength class of C30/37 [5]. In addition, the specimen’s aggregate grading curve (sieve line), which is the key parameter for ultrasonic wave scattering, is analogous to that of the other two FOR 384 concrete step specimens. The grading curve is illustrated in detail in Fig. 7 (green solid line).

**Fig. 8:**
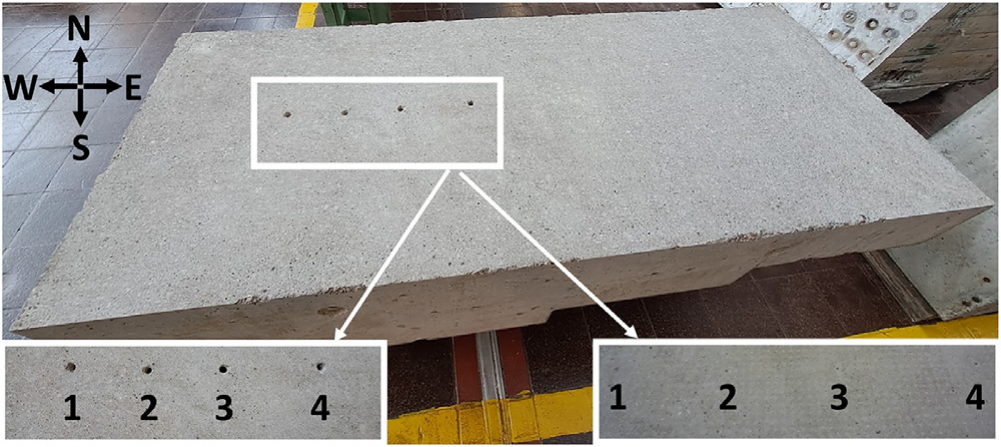
Four boreholes on the surface of the concrete specimen – before (left) and after (right) grouting with mortar.

Steel reinforcement is incorporated into the specimen to prevent the formation of cracks in the concrete. In addition, four cuboidal built-in elements made of polystyrene rigid foam are embedded in the specimen, one cuboid in each step (Fig. 10). Each cuboid has dimensions of 120 × 120 × 60 mm. These elements are incorporated into the specimen to simulate material irregularities in concrete constructions such as voids or built-in elements like rectangular tendon ducts. The exact positions of the polystyrene rigid foam cuboids are outlined in the technical drawings in Appendix A. Furthermore, upon delivery from MPA Stuttgart to BAM, the concrete specimen had four non-documented boreholes on its surface (Fig. 8). These boreholes were filled at BAM with grouting mortar (CEM I 42,5 R (ep)) (Fig. 8). Table 1 provides details on their respective depths, diameters, and inclinations. The exact locations of the boreholes are documented in the technical drawings in Appendix A.

**Table 1:** Details on the depths, diameters, and inclinations of the four boreholes.

Structural Element	Depth [mm]	Diameter [mm]	Inclination to the west [°]	Inclination to the north [°]
Borehole No. 1	80	14	1	2
Borehole No. 2	86	14	1	1
Borehole No. 3	69	14	0	0
Borehole No. 4	64	14	2	0

**Fig. 9:**
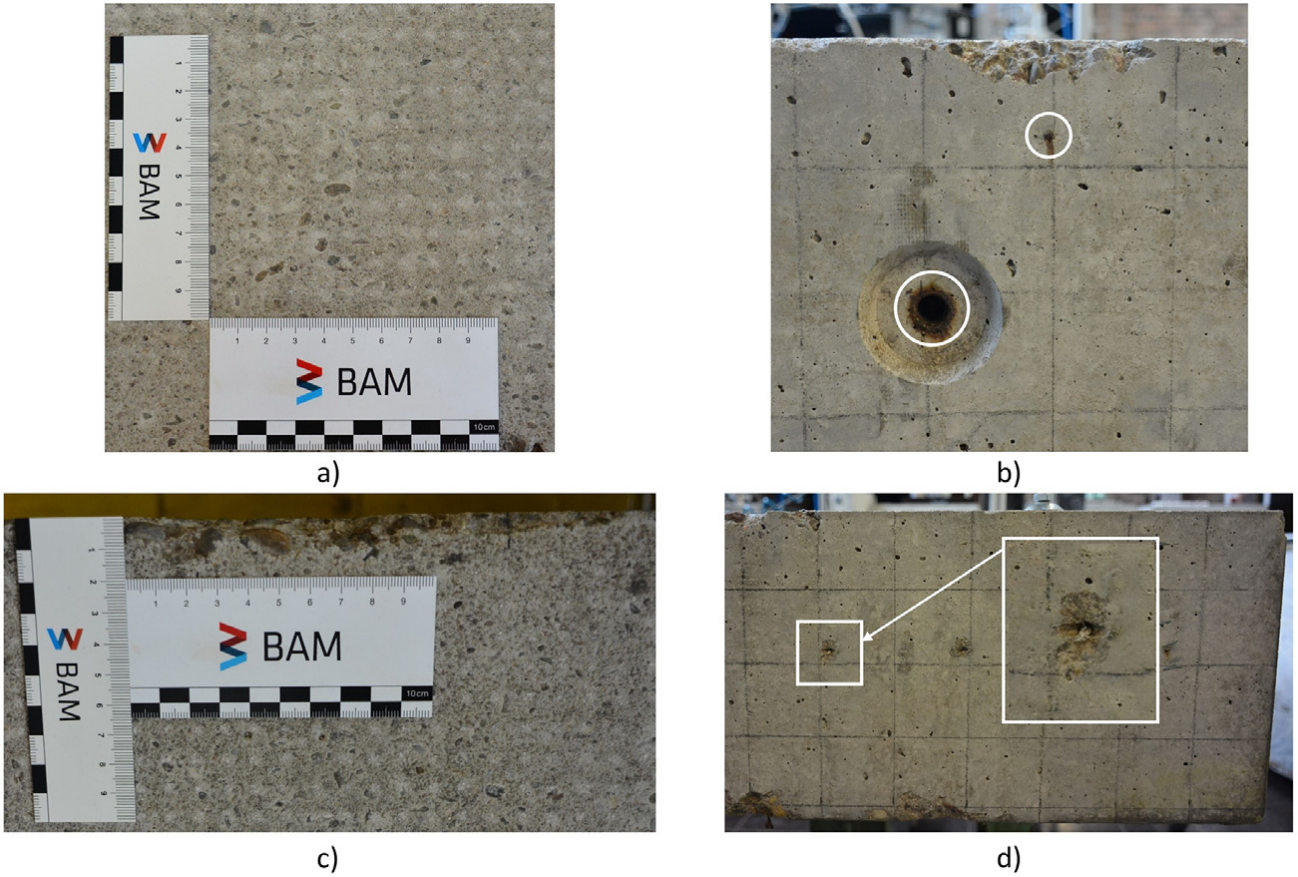
Concrete specimen Pk401: a) detailed view of the mechanically processed surface, b) reinforcement indicated by white circles, c) spalling along the edges, and d) fishing lines used to secure the built-in elements made of polystyrene rigid foam – one line is highlighted exemplarily with a white rectangle.

The concrete specimen has a slightly rough surface, suggesting that the cement matrix has been mechanically processed (Fig. 9a). The surface was lightly sanded (≈ 1 mm removal) to remove slurry and small irregularities, probably introduced when the fresh concrete was smoothed by hand. This light sanding evened the surface without materially altering the specimen. As a result, the surface of the concrete specimen does not provide the typical contact conditions for the ultrasonic probes usually encountered in NDT of concrete structures. Nevertheless, this had no adverse effect on the quality of the ultrasonic signals and the ultrasonic raw data show no loss of detectability for the embedded built-in elements or the back wall of the specimen. Moreover, the specimen’s surface exhibits partial spalling along the edges (Fig. 9c).

**Fig. 10:**
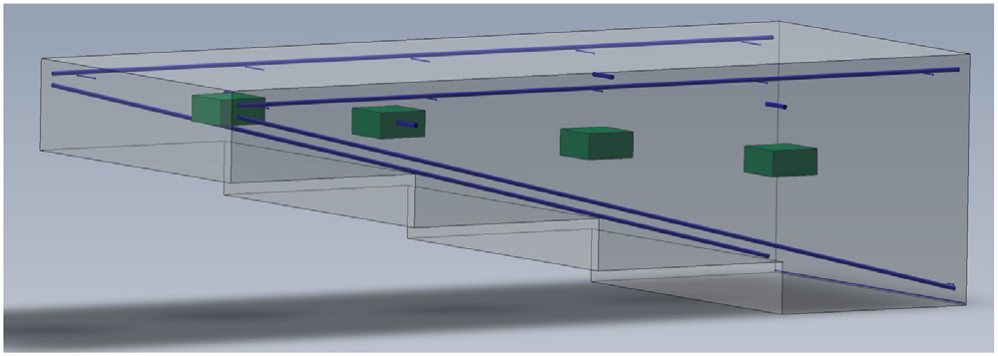
3D model of the concrete specimen Pk401 with four embedded polystyrene rigid foam cuboids (green).

Fig. 10 demonstrates a 3D model of the concrete specimen, while the technical drawings in the appendices (Appendix A and Appendix B, Appendix A and Appendix B) provide detailed geometric dimensions of the specimen, including both lateral and top views. The technical drawings in Appendix A illustrate the specimen without reinforcement, while the technical drawings in Appendix B show it with reinforcement. The reinforcement is positioned in the edge areas of the concrete specimen and serves not only to prevent cracks but also to secure the position of the built-in elements. Additionally, due to the distance between the reinforcement and the built-in elements, its influence on the ultrasonic measurements is expected to be minimal. The reinforcement details shown in Appendix B are approximate values, determined through ground penetrating radar measurements. Only for the reinforcement visible on the outer sides of the specimen (marked blue and black in Appendix B) an exact diameter could be specified (exemplarily shown in Fig. 9b). The cuboidal built-in elements made of polystyrene rigid foam were each secured with two fishing lines (synthetic material) before concreting; the lines are visible along the outer edges (as exemplarily shown in Fig. 9d).

According to the technical drawings, the X-Y plane at Z=0 corresponds to the measuring plane, namely the upper component surface. The specified thicknesses (dimensions in Z-direction) of the individual component steps represent average values, calculated from twelve thickness measurements taken at each respective step. The standard deviations of the twelve thickness measurements for each respective step are presented in Table 2. The dimension of the concrete specimen in Y-direction is 796.4 mm, which represents the average value of 8 individual measurements. The corresponding standard deviation is ± 0.54 mm. All sides of the concrete specimen exhibit a production-related imperfection of about ± 1 mm. Furthermore, the technical drawings in Appendix A show the exact positions of the cuboidal built-in objects made of polystyrene rigid foam, according to the construction plans from MPA Stuttgart. A detailed verification of the positions of these embedded elements was not technically possible at BAM. Therefore, minor positional deviations may have occurred during concreting. The technical drawings in Appendix A also illustrate the exact X and Y coordinates of the four mortar-filled boreholes.

**Table 2:** Thicknesses of the four steps of the concrete specimen with indication of the standard deviation.

Structural Element	Depth in Z-direction [mm]
Step No. 1	573,8 ± 0,66
Step No. 2	453,4 ± 0,83
Step No. 3	333,1 ± 0,82
Step No. 4	210,4 ± 0,89

### 4.2 Ultrasonic measurement equipment

**Fig. 11:**
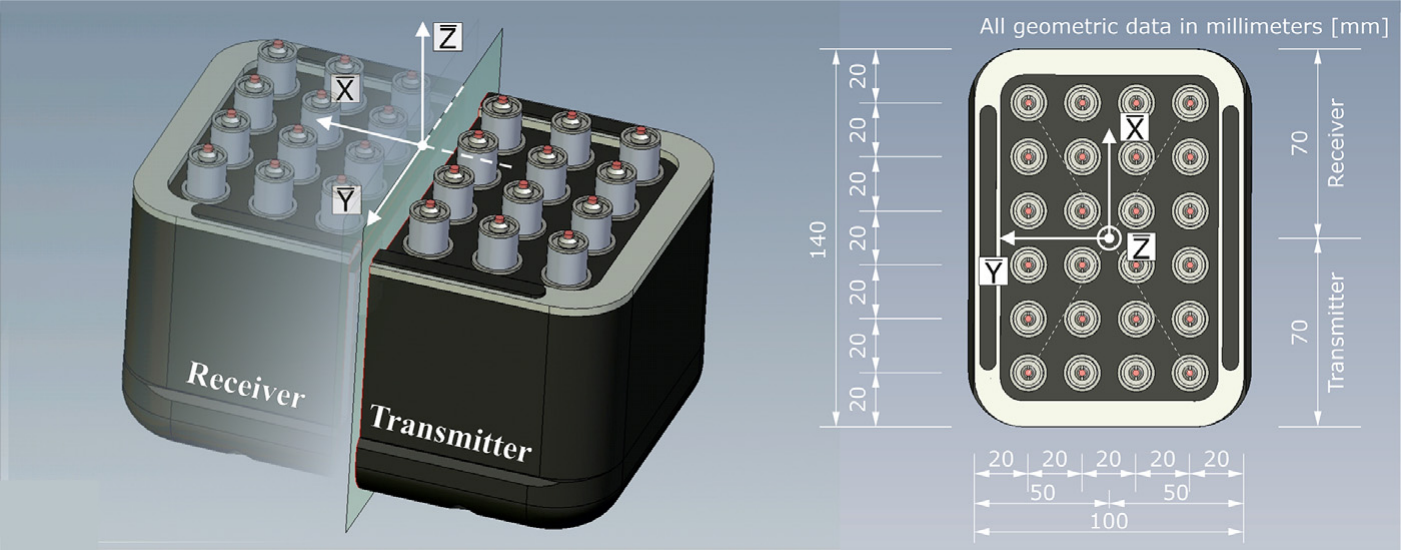
Geometrical dimensions of the probes used for transmitting and receiving longitudinal waves (M2503) and shear waves (SH) (M2502), extracted from [2].

The ultrasonic measurements were conducted using probes with identical geometry for both wave types (M2503 for longitudinal waves and M2502 for shear waves). Both ultrasonic probes were custom-made by Acoustic Control Systems ACS [7] for BAM. Fig. 11 illustrates the geometric dimensions of the probes, along with the local coordinate system ($\bar{X},\;\bar{Y},\;\bar{Z}$), whose origin is located at the center of the aperture. Each probe consists of an array of 24 spring-mounted dry point contact transducers, with 12 acting as transmitters and the other 12 as receivers, creating a monostatic arrangement [3]. The dry point contact transducers are highly damped broadband transducers (type S1802 for shear [8] and type S1803 [9] for longitudinal). Within each unit, 12 individual transducers are connected in parallel.

For the ultrasonic measurements, the probes must be attached to the concrete surface by applying soft pressure, and the acoustic pulse is inserted into the concrete via the piezoelectric effect. The diameter of each transducer's circular contact surface is approximately 1.7 mm.

The longitudinal wave generated by the M2503 probe is polarized in the $\bar{Z}$-direction in relation to the coordinate system shown in Fig. 11 and is excited with a frequency of f = 80 kHz (the nominal frequency is 100 kHz). The shear wave generated by the M2502 probe is polarized horizontally (SH wave) in the $\bar{Y}$-direction in relation to the coordinate system shown in Fig. 11. The SH waves are excited with a frequency of f = 50 kHz (the nominal frequency is 50 kHz). Both probes have a bandwidth of approximately 60–70% [10].

**Fig. 12:**
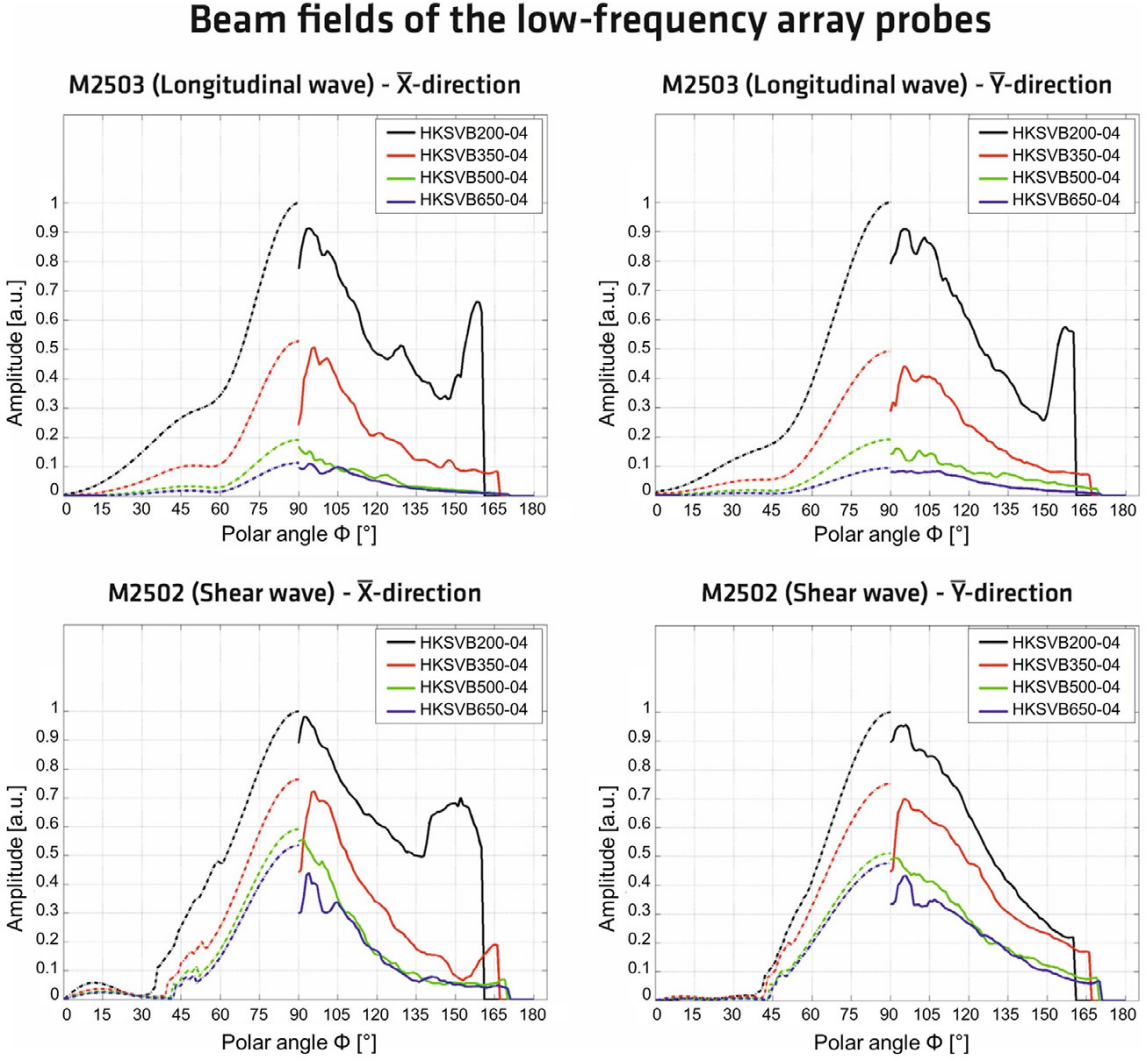
2D representations of the directivity patterns of the probes M2502 and M2503, with solid lines indicating the results of measurements on concrete hemispheres measuring 200 mm, 350 mm, 500 mm, and 650 mm in diameter. The dashed lines represent the results of simulations. The radiation pattern was determined for concrete with a maximum aggregate size of 4 mm (label -04 in the legend) and for different distances from the ultrasonic source (200 mm, 350 mm, 500 mm, and 650 mm). The results were extracted from [2], which are based on the results published in [11].

The arrangement of the 12 transmitting point contact transducers produces geometrically focused wavefields, whose characteristics are shown in Fig. 12 for both wave types and different planes. Fig. 12 displays measurement (solid lines) and simulation results (dashed lines) for the respective principal axes $\bar{X}$ and $\bar{Y}$ (see coordinate system in Fig. 11). The measurements and simulations were conducted at varying distances from the surface where the sound pulse was generated [2]. The simulation and measurement procedures together with a detailed comparison of both are fully documented in Spies et al. [11]. Further information on the measurements and the measurement technique is provided in Maack et al. [12]. The key distinction between the results in Fig. 12 and the data obtained from the concrete specimen is the aggregate size: the concrete mixture used to characterise the wavefields contains aggregates with a maximum diameter of 4 mm [2], whereas the specimen analysed here uses 16 mm.

**Fig. 13:**
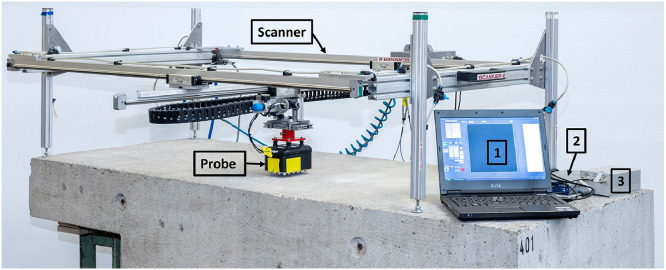
Automated scanner system and additional electronic measurement equipment used for the ultrasonic echo measurements (1: measuring laptop, 2: DAQ-Pad and 3: square wave pulser).

The ultrasonic echo measurements with the probes described above were performed using a fully automated scanner system developed at BAM (Fig. 13). Fig. 13 further illustrates the additional electronic measurement equipment used. It consists of a measuring laptop (1), a data acquisition (DAQ)-Pad from National Instruments (NI USB-6361) (2) and a square wave pulser (transmission voltage: ±160 V) (3) for generating the source signal. The latter was self-constructed at BAM. A receiver amplifier is integrated inside the probes. A LabView program (also developed at BAM) installed on the measuring laptop was used to start the measurement, to control the scanner as well as to record and store the ultrasonic data. From the DAQ-Pad, a trigger signal was sent to the square-wave pulser, and the recording of the ultrasonic data was started at the same time. After receiving the trigger signal, the square-wave pulser sent a square-wave signal to the transmitting transducer array. This source signal was converted into a sinusoidal-like oscillation due to the characteristic properties of the transducers. The signal received by the receiving transducer array was then forwarded to the measuring laptop via the DAQ-Pad after amplification by the receiver amplifier. The measurement technique used produced a time delay in the measurement data, which may need to be determined and considered during the evaluation of the ultrasonic data, depending on the specific task.

### 4.3 Measurement setup and process

**Fig. 14:**
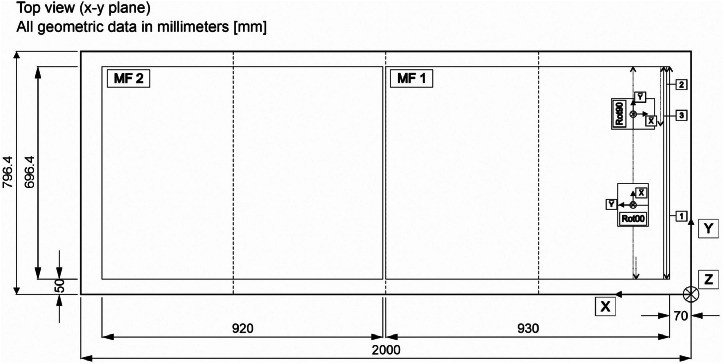
Overview of the measurement fields MF 1 and MF 2 with the applied probe orientations (Rot00 and Rot90). Measurement lines 1 to 3 are highlighted as examples.

To collect the ultrasonic data, the automated scanner system (Fig. 13) scanned the surface of the concrete specimen in a meandering pattern along a predefined measurement grid. In Fig. 14, the first three measurement lines (labelled 1 – 3) are indicated exemplarily by black lines. The probe was moved along the Y-direction. The measurement point spacing in both the X- and Y-directions was 10 mm. During the transition between the measurement points, the probe was temporarily removed from the concrete surface and then pressed against it at each measurement point without any couplant to transmit the acoustic pulse and record the ultrasonic data. Fig. 14 illustrates the two different probe orientations used which were denoted in the datasets with Rot00 and Rot90. For the orientation Rot00 the $\bar{X}$ – axis of the probe is aligned along the measurement direction (Y-direction), whereas for the orientation Rot90 the $\bar{Y}$ - axis of the probe is aligned along the measurement direction.

Since the length of the specimen in the X-direction exceeds the scanning system's range, the upper surface of the concrete specimen was divided into two measurement fields (Fig. 14). Measurement field 1 (MF 1) has a length of X_MF1_ = 930 mm, while measurement field 2 (MF 2) measures X_MF2_ = 920 mm. These fields were subsequently digitally merged into a single measurement field and stored.

In order to mount the entire probe on the surface of the concrete specimen, it was necessary to maintain a distance of 70 mm in X-direction and 50 mm in Y-direction from the outer edges of the specimen to the centre of the probe (Fig. 14). To ensure that the measurement area size matches the surface dimensions of the concrete object, measurement points outside the measurement fields MF 1 and MF 2 (non-actual measurement points) were replaced with zero vectors in the measurement data (see section DATA DESCRIPTION). Further measurement parameters and additional information about the data acquisition are presented in Table 3 for the four different datasets.

**Table 3:** Information about measurement parameters and data acquisition. The positions marked with * in the entries in column No. 1 (Dataset) vary between the datasets.

Dataset	Probe type	Measurement frequency [kHz]	Size measurement field (X x Y)	Grid spacing [mm]	Samples (per A-Scan) []	Sampling frequency [MHz]	Amplitude range [mV]
*Long*Rot00*	M2503	80	1860 mm × 700 mm	10	4000	2	± 1000
*Long*Rot90*	M2503	80	1860 mm × 700 mm	10	4000	2	± 1000
*Shear*Rot00*	M2502	50	1860 mm × 700 mm	10	4000	2	± 2000
*Shear*Rot90*	M2502	50	1860 mm × 700 mm	10	4000	2	± 2000

## Limitations

**Fig. 15:**
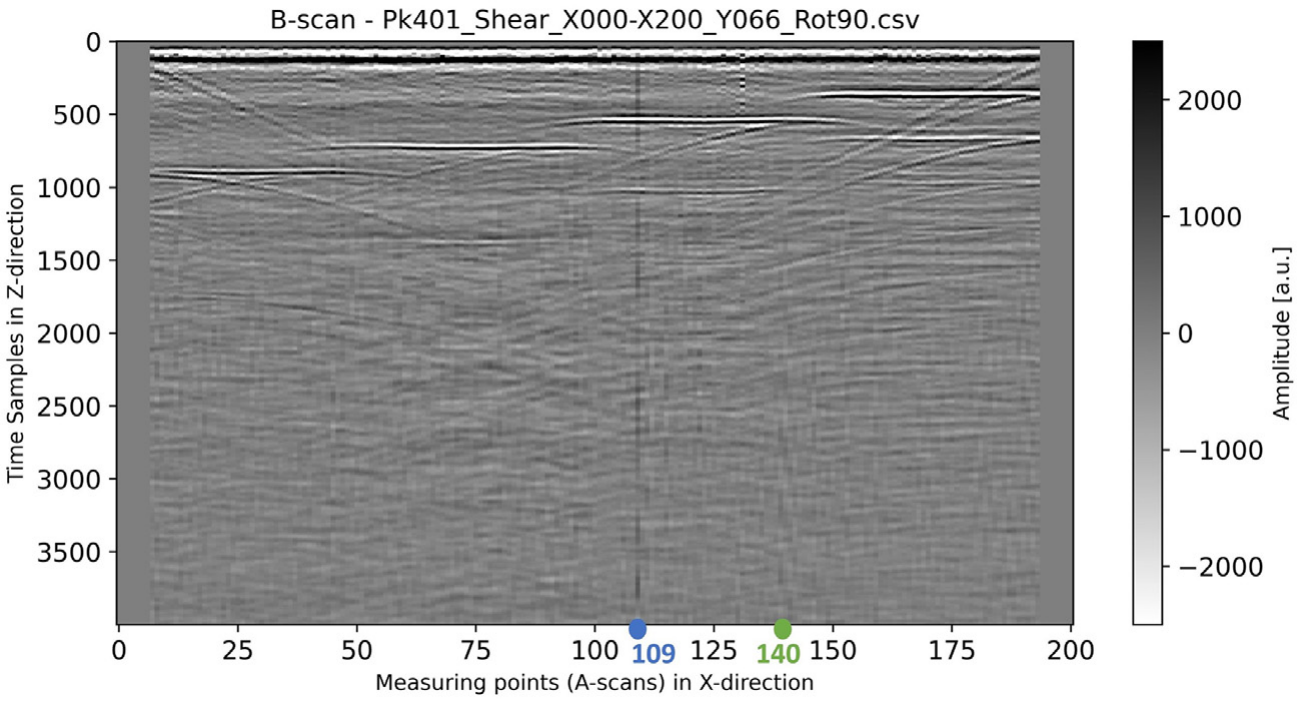
B-scan (color-coded in grayscale) generated from the dataset “Pk401_Shear_X000-X200_Y066_Rot90.csv”. The green and blue circles mark the positions of the A-scan No. 109 and No. 140, which are shown in Fig. 16.

**Fig. 16:**
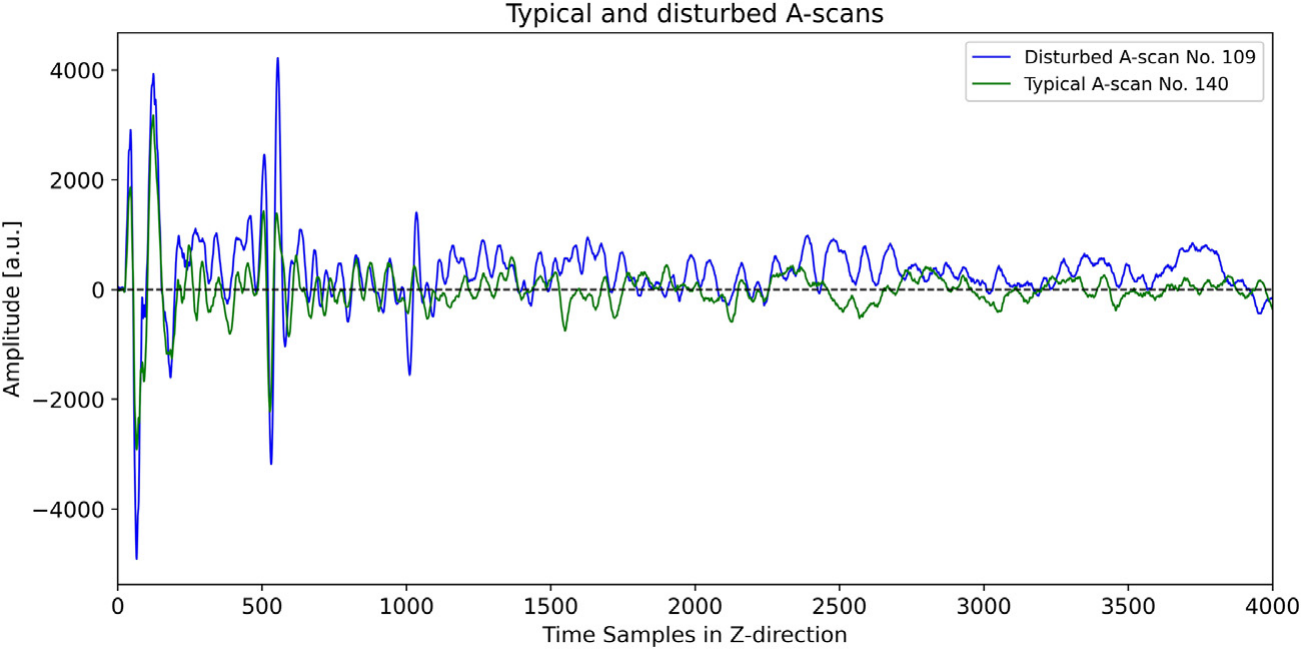
Typical (green) and disturbed (blue) ultrasonic signals (A-scans), extracted from the B-scan shown in Fig. 15.

Fig. 15 shows a B-scan generated from the CSV dataset *Pk401_Shear_X000-X200_Y066_Rot90.csv*. Fig. 16 illustrates two corresponding A-scans (No. 109 and No. 140). The A-scan No. 140 shows a healthy typical ultrasonic signal whereas the A-scan No. 109 is affected by a DC offset, which shifts it away from the zero line at around sample 1000. In some other B-scans this DC offset becomes noticeable earlier than the first 1000 samples. This observed disturbance can be caused either by the measurement setup (e.g. the receiver amplifier) or by external electromagnetic interference. The exact source cannot be identified. Fortunately, only a few A-scans are affected by this DC offset, and it can be eliminated during post-processing using digital filters.

**Fig. 17:**
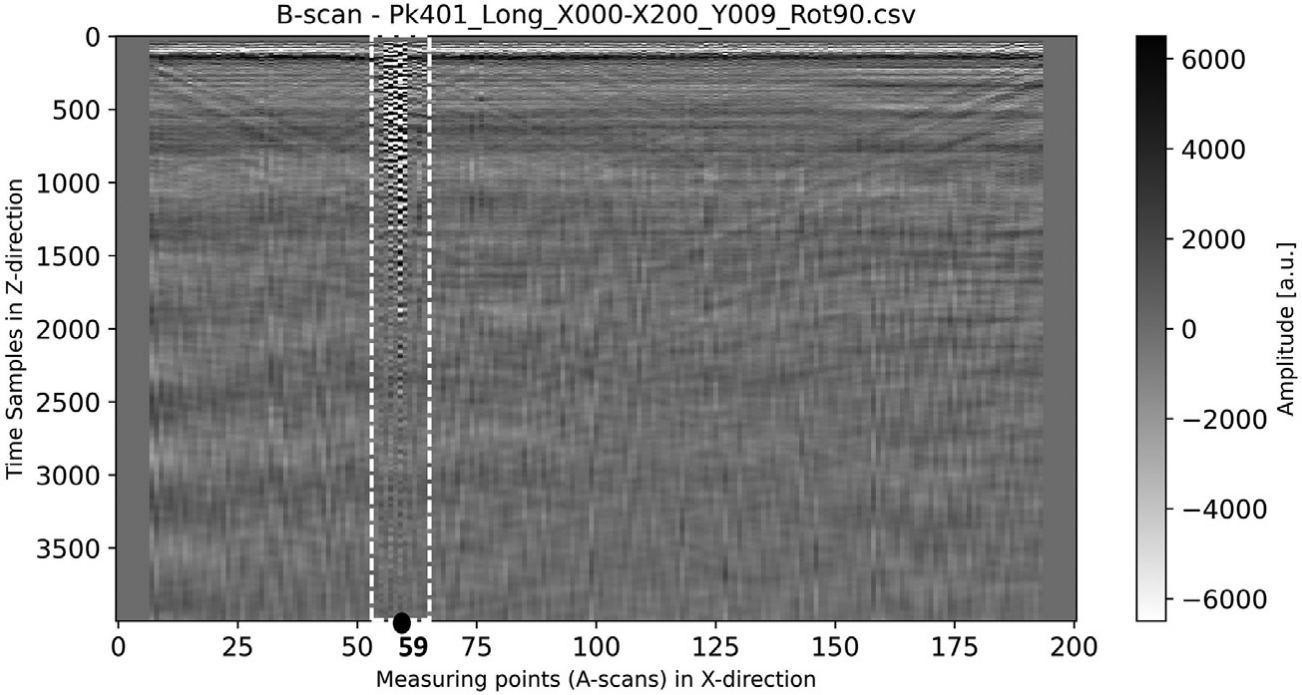
B-scan No. 9 (color-coded in grayscale) generated from the dataset “Pk_401_3D_Dataset_Long_Wave_Rot90.npy” (corresponds to the dataset Pk401_Long_X000-X200_Y009_Rot90.csv). The black circle marks the position of the A-scan No. 59, which is shown in Fig. 18. The white dashed rectangle marks the area of the A-scans No. 53 to 63.

**Fig. 18:**
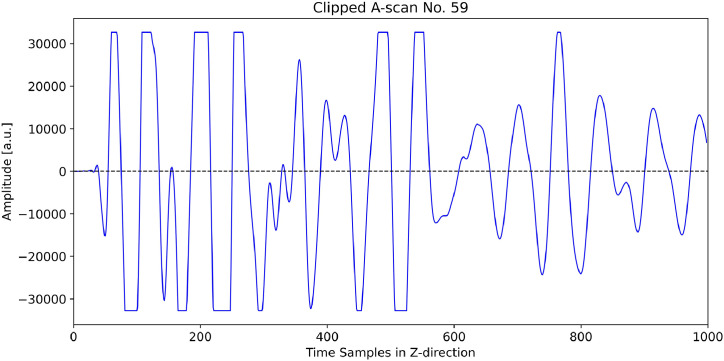
Clipped A-scan extracted from the B-scan shown in Fig. 17.

Furthermore, across all four 3D datasets, disturbed ultrasonic signals are visible in approximately B-scans No. 5 to 11 in the area of the A-scans No. 53 to 63. The cause of these disturbances is not visible from the outside. While we do not know its exact origin, we can say with certainty that it arises in the near-surface region. As a result, some A-scans are clipped, but this only occurs in the longitudinal wave datasets. Fig. 17 exemplarily shows B-scan No. 9, generated from the dataset *Pk_401_3D_Dataset_Long_Wave_Rot90.npy*, and Fig. 18 illustrates the corresponding A-scan No. 59. The area of disturbance is highlighted in the B-scan with a white rectangle. The signal in Fig. 18 is clearly clipped at the beginning because the acquisition electronics reached saturation. This clipping could not be prevented, even by increasing the amplitude range of the measurement system.

**Fig. 19:**
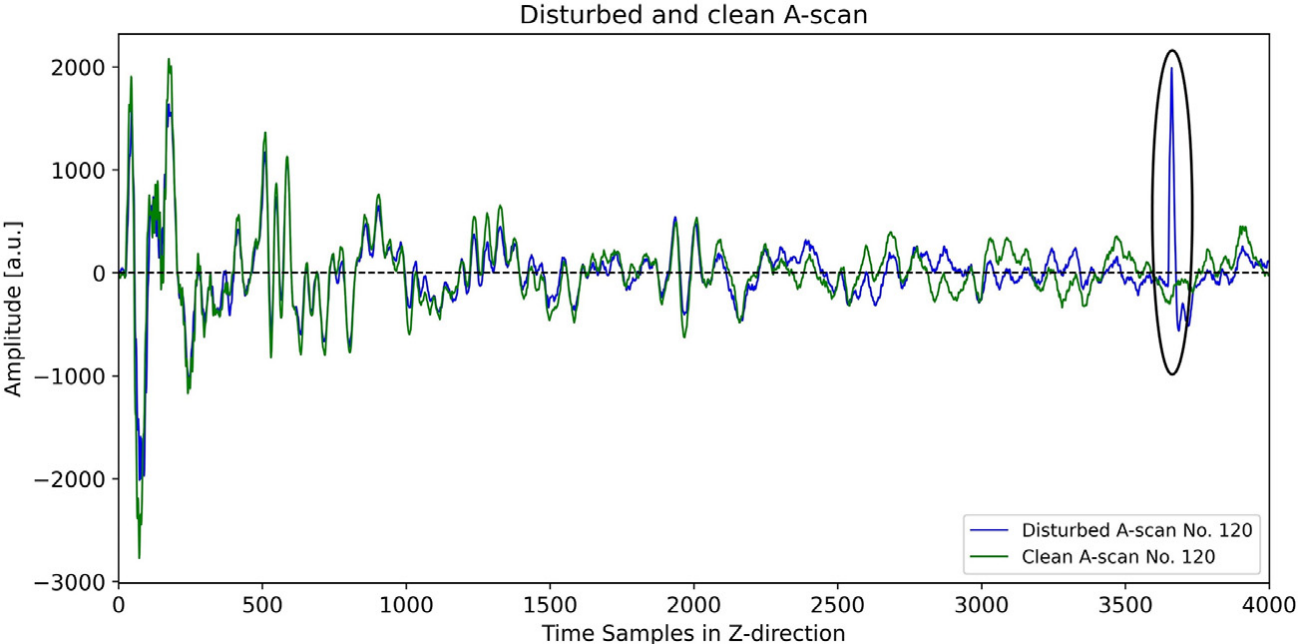
Disturbed (blue) and clean (green) A-scan No. 120, both extracted from the corresponding B-scan No. 74 of the shear wave datasets (probe orientation Rot90). The disturbance is highlighted with a black circle. The clean A-scan (green) was extracted from the dataset Pk401_Shear_X000-X200_Y074_Rot90.csv.

Moreover, for the data acquired with the probe orientation Rot00, the probe was not perfectly positioned on the surface for the first and last B-scan (B-scan No. 5 and No. 75) of the measurement area. For the B-scan No. 5, one row (four dry-point contact transducers) of the receiving transducer array was positioned outside the concrete specimen, while for B-scan No. 75, one row of the transmitting transducer array was positioned outside the specimen. This occurred because the specimen's Y-dimension is 796.4 mm rather than 800 mm, as in the two other concrete specimens from Maack et al. [2]. Nevertheless, we chose to use 71 measurement points in Y-direction and hence, to retain B-scans No. 5 and 75, to ensure consistency with the data published in Maack et al. [2]. Their inclusion reflects reality as probe coupling is not always ideal in practice. Furthermore, both B-scans have no adverse effect on the detectability of the specimen’s back wall or embedded built-in elements. Additionally, some transducers of the probes did not achieve perfect coupling with the concrete specimen at locations where spalling occurred along the edges of the concrete object (see section CONCRETE SPECIMEN Pk401, Fig. 9c), a situation that is often encountered in real-world NDT scenarios. Because the dry-point contact transducers are spring mounted (Fig. 11), they still applied enough pressure at these locations. A review of the raw ultrasonic data showed no loss of amplitude there. Thus, the minor coupling deviations did not affect the recorded ultrasonic signals. During the measurements, there were occasional disturbances in the signals caused by interference from the power grid. Although these were significantly reduced by using an isolating transformer, they could not be completely prevented. For this reason, each ultrasonic measurement was repeated once, and the disturbed signals were replaced by clean signals. Fig. 19 exemplarily shows, the disturbed A-scan No. 120 (blue) and the clean A-scan No. 120 (green) from the repeated measurement, both extracted from the corresponding B-scan No. 74 of the shear wave datasets (probe orientation Rot90). The clean A-scan No. 120 was extracted from the dataset *Pk401_Shear_X000-X200_Y074_Rot90.csv*. Table 4 shows the positions of the disturbed A-scans in all four datasets which were replaced by clean signals.

**Table 4:** Positions of the disturbed ultrasonic signals (A-scans) within the corresponding B-scans, which were replaced by clean signals from the repeated measurements.

Dataset	B-scan No.	A-scan No.
*Shear*Rot00*	21	80
	23	150
	38	128
	72	139
*Shear*Rot90*	17	118
	37	180
	40	101
	43	53
	47	142
	52	78
	64	127
	65	192
	70	66
	74	120
*Long*Rot00*	10	31
	55	181
	71	124
*Long*Rot90*	6	23
	12	71 & 100
	21	82
	31	156
	47	106
	71	94
	75	77

## Ethics Statement

The authors have read and follow the ethical requirements for publication in Data in Brief, and confirm that the data do not involve human subjects, animal experiments, or any data collected from social media platforms.

## CRediT authorship contribution statement

**Maria Grohmann:** Conceptualization, Methodology, Investigation, Software, Formal analysis, Writing – original draft, Data curation. **Stefan Maack:** Methodology, Formal analysis, Writing – review & editing. **Matthias Behrens:** Visualization. **Ernst Niederleithinger:** Supervision, Writing – review & editing.

## Data Availability

DataverseLow-frequency ultrasound data (pulse-echo technique) of shear horizontal and longitudinal waves acquired on the concrete step specimen ``Pk401'' with embedded polystyrene foam cuboids (Original data).
